# Innovative optimization of greater omentum imaging report and data system for enhanced risk stratification of omental lesions

**DOI:** 10.1186/s40644-025-00848-2

**Published:** 2025-03-10

**Authors:** Zhiguang Chen, Liang Sang, Yuan Cheng, Xuemei Wang, Mutian Lv, Yanjun Liu, ZhiQun Bai

**Affiliations:** 1 Department of Ultrasound, The First Hospital of China Medical University, No. 155, Nanjing North Street, Heping District, Shenyang, Liaoning Province 110001 China; 2 Department of Nuclear Medicine, The First Hospital of China Medical University, No. 155, Nanjing North Street, Heping District, Shenyang, Liaoning Province 110001 China

**Keywords:** The greater omentum, Omental score, Contrast-enhanced ultrasound, Greater omentum Imaging-Reporting and data system, Real-time elastography

## Abstract

**Background:**

In 2020, we introduced the Greater Omentum Imaging-Reporting and Data System (GOI-RADS), a novel classification system related to peritoneal lesions. However, its clinical application remained unvalidated.

**Objective:**

This study aimed to validate GOI-RADS, optimize its parameters for a new grading system, and explore its clinical usefulness.

**Methods:**

A retrospective-prospective study was conducted to validate and refine the GOI-RADS system. The study consisted of two phases: a retrospective validation phase and a prospective application phase. The first phase included patients with peritoneal lesions from 2019 to 2021, classified by GOI-RADS and verified against pathology. Contrast-enhanced ultrasound (CEUS) and real-time elastography (RTE) data were collected for developing a new grading system. Odds ratios optimized parameters. The second phase (2021–2024) assessed diagnostic consistency among sonographers and performance of grading systems.

**Results:**

Among 215 patients with peritoneal lesions, the actual malignancy rates for GOI-RADS 2 (40.00%) and GOI-RADS 3 (61.22%) were much higher than predicted (5.56% and 37.25%). Combining CEUS and RTE parameters showed varying sensitivity and specificity: RTE + GOI-RADS (95.35%, 55.56%) and CEUS + GOI-RADS (96.51%, 44.44%). However, the grading system based on multiple ultrasound parameters, specifically when incorporating RTE, CEUS parameters, and GOI-RADS (Multi-GOIRADS), exhibited the highest diagnostic sensitivity and specificity of 88.37% and 83.33%, respectively. Its simplified version, sMulti-GOIRADS, had sensitivity of 73.26% and specificity of 94.44%. In the prospective study involving three sonographers of different qualifications, the use of sMulti-GOIRADS was found to be the most time-efficient and showed excellent diagnostic consistency among them. In contrast, Multi-GOIRADS required more time for scoring but offered superior diagnostic performance, particularly among senior sonographers (88.35% and 91.43%).

**Conclusions:**

This study proposes a multiparametric ultrasound-based imaging-reporting and data system for risk stratification of omental malignancy, Multi-GOIRADS, and presents an optimized and simplified version, sMulti-GOIRADS, which demonstrates excellent diagnostic consistency and performance in clinical applications.

**Supplementary Information:**

The online version contains supplementary material available at 10.1186/s40644-025-00848-2.

## Introduction

The peritoneum, the largest and most intricate serous membrane in the human body, consists of mesothelial cells interspersed with a minimal amount of connective tissue. It encompasses the parietal peritoneum, greater omentum, lesser omentum, mesentery, and various ligaments [1, 2]. Notably, the greater omentum stands out as the largest peritoneal fold and is frequently the initial site for peritoneal metastasis of cancer cells [3]. Over the past decade, there has been a surge in the number of patients diagnosed with peritoneal metastatic cancer, often at an advanced stage. Although some patients may undergo cytoreductive surgery combined with hyperthermic intraperitoneal chemotherapy, high rates of postoperative recurrence and complications persist [4–7]. Consequently, early detection of metastatic cancer in the greater omentum and timely clinical intervention have emerged as key areas of research focus. However, the current landscape lacks effective imaging modalities for predicting and managing greater omental lesions.

In 2020, Chen et al. [8] introduced the Greater Omentum Imaging Reporting and Data System (GOI-RADS), which categorizes greater omental lesions into four classes based on their scores and associated malignancy rates. Specifically, GOI-RADS 1 indicates a score of ≤ 5 with a malignancy rate of 0%; GOI-RADS 2 corresponds to a score of 6–7 with a malignancy rate of ≤ 5.56%; GOI-RADS 3 has a score of 8–9 with a malignancy rate of ≤ 37.25%; and GOI-RADS 4 has a score of ≥ 10 with a malignancy rate of ≥ 87.72%. This system aims to provide sonographers with a valuable reference for diagnosing peritoneal diseases. However, to date, no studies have evaluated the clinical application effectiveness of this system. Therefore, the objective of this study is to investigate the clinical application value of GOI-RADS by integrating ultrasound elastography and contrast-enhanced ultrasound (CEUS) results. This integration aims to expand GOI-RADS and adjust the weighting parameters to enhance the omental scoring process. We propose a new grading system and validate its diagnostic effectiveness through a prospective study. Ultimately, our goal is to facilitate the clinical application and promotion of the refined greater omentum grading system.

## Materials and methods

The Ethics Committee of the First Affiliated Hospital of China Medical University approved the study protocol (NO. AF-SOP-07-1.2-01).

### Patients

**Fig. 1:**
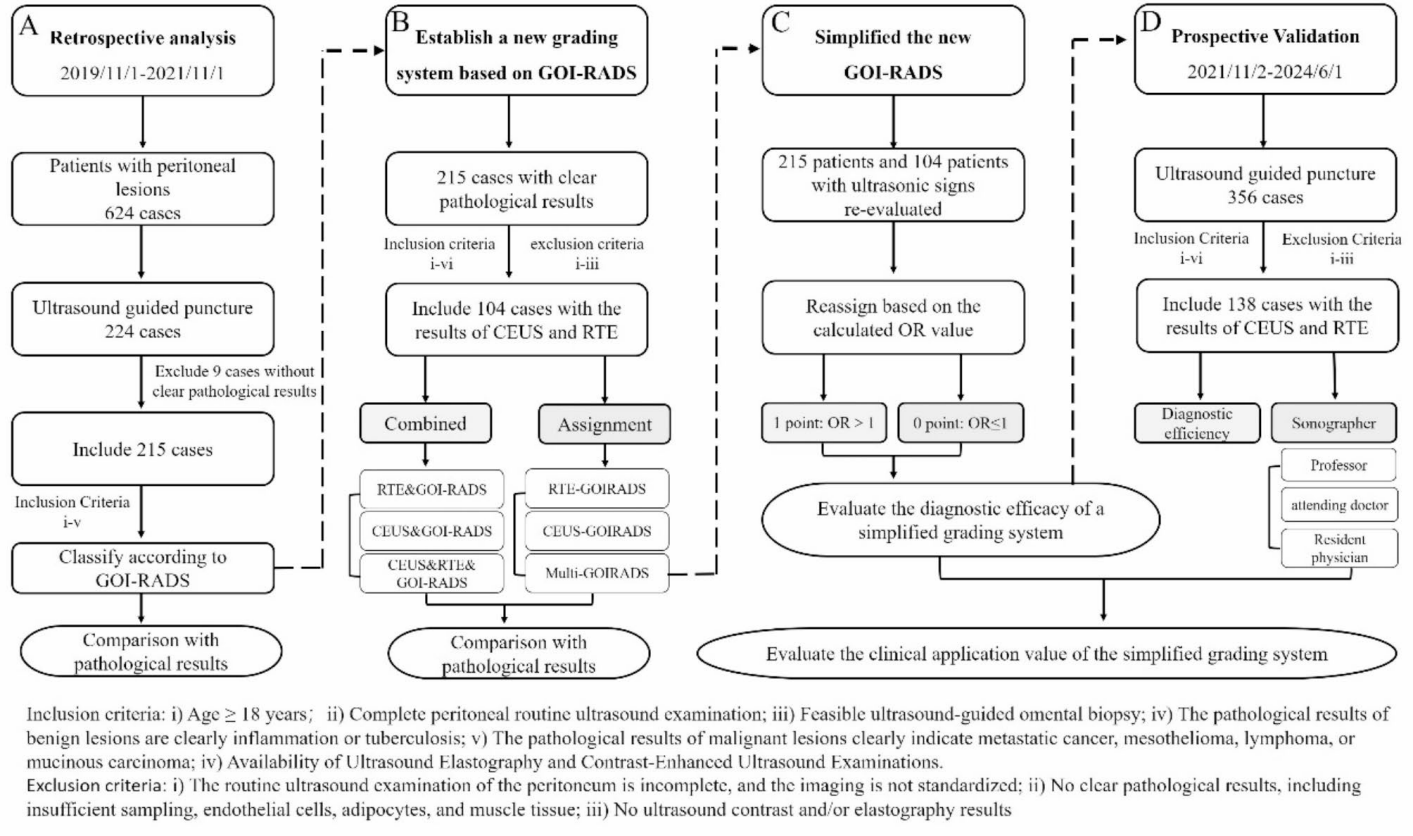
Experimental Roadmap and Inclusion/Exclusion Criteria. (**A**) Retrospective analysis of clinical applicability and efficacy of GOI-RADS; (**B**) Development of a new grading system based on CEUS and RTE findings; (**C**) Simplified the established newly grading system; (**D**) Prospective validation of the clinical value of the new grading system

This study is a retrospective analysis coupled with a prospective validation. For the retrospective analysis, we collected data from patients with peritoneal lesions who consecutively visited our facility from November 2019 to November 2021. The patients were categorized based on the GOI-RADS. Patients eligible for omentum biopsy (see supplementary materials) underwent ultrasound-guided omentum puncture biopsy. All patients signed informed consent forms prior to the procedure. The diagnostic concordance rate and accuracy of GOI-RADS were verified against pathological findings. For patients who completed CEUS and real-time elastography (RTE), a multimodal ultrasound combined diagnosis or a new grading system incorporating CEUS, RTE, and GOI-RADS was developed. For the prospective validation phase, we recruited patients with peritoneal lesions who consecutively visited our facility from November 2021 to June 2024. These patients were evaluated according to the newly established classification system, and the diagnostic consistency among sonographers with varying levels of experience was studied. The optimal classification system was selected for widespread application (Fig. 1).

The inclusion and exclusion criteria are illustrated in Fig. 1.

### Ultrasound examination

#### Ultrasonic instruments

Routine ultrasound examination and RTE: Canon Aplio i900 color Doppler ultrasound diagnostic apparatus (Canon Medical Systems Co., Ltd., China), with high-frequency linear array probe i18LX5 and low frequency convex array probe i8CX1.

Contrast enhanced ultrasound and ultrasound-guided puncture biopsy: Mindray, Resona 8EXP color Doppler ultrasound diagnostic apparatus (Shenzhen, China), with high-frequency linear array probe L12-3E and low-frequency convex array probe C5-2E.

#### Routine ultrasound examination and RTE

Patients are instructed to fast for 8 h prior to the examination. They are positioned in a supine position with their abdomen fully exposed. A comprehensive abdominal scan is performed using a low-frequency convex array transducer (i8CX1). Key parameters recorded include omental thickness, echogenicity, ascites echogenicity, presence of septations, and visibility of mesenteric lymph nodes. And then switched to the high-frequency linear array transducer (i18LX5) for closer observation. The omental structure and the presence of parietal peritoneum are carefully examined. Based on the GOI-RADS, suspicious areas within the omentum are identified. Patients are instructed to exhale and hold their breath. The RTE imaging conditions are activated. The imaging region is adjusted according to the location of the pathological omentum. A stable and continuous pressure is manually applied to the transducer. Once the image is fully filled and stabilized, it is captured and stored for further analysis.

#### CEUS and ultrasound-guided puncture biopsy

For patients eligible for CEUS and ultrasound-guided puncture biopsy, they were informed of potential complications before undergoing the procedures. All patients signed informed consent forms and were accompanied by family members during the examination. For patients undergoing puncture biopsy, they were required to stay for observation for at least 12 h after the procedure and could only leave when they felt physically well. CEUS Procedure: A low-frequency convex array transducer was used for a comprehensive abdominal scan. The transducer was switched to CEUS mode at the intended puncture site, and CEUS imaging parameters were adjusted accordingly. A 1.5-2mL dose of SonoVue contrast agent was injected via the elbow vein, followed by a rapid bolus of 5mL saline. The start time, peak time, and washout time of contrast enhancement in the lesioned omentum were recorded. The entry and washout patterns of the contrast agent (fast-in/iso-in/slow-in, fast-out/iso-out/slow-out) were noted in comparison with the surrounding tissues. Additionally, the enhancement pattern (homogeneous or heterogeneous enhancement) was documented. Based on the findings from conventional ultrasound and CEUS, the puncture path was selected, and the transducer was switched to a high-frequency linear array for real-time guidance. The needle was inserted obliquely along the abdominal wall incision, and three omentum tissue samples from different levels were obtained for pathological examination.

### Clinical application research of GOI-RADS

**Fig. 2:**
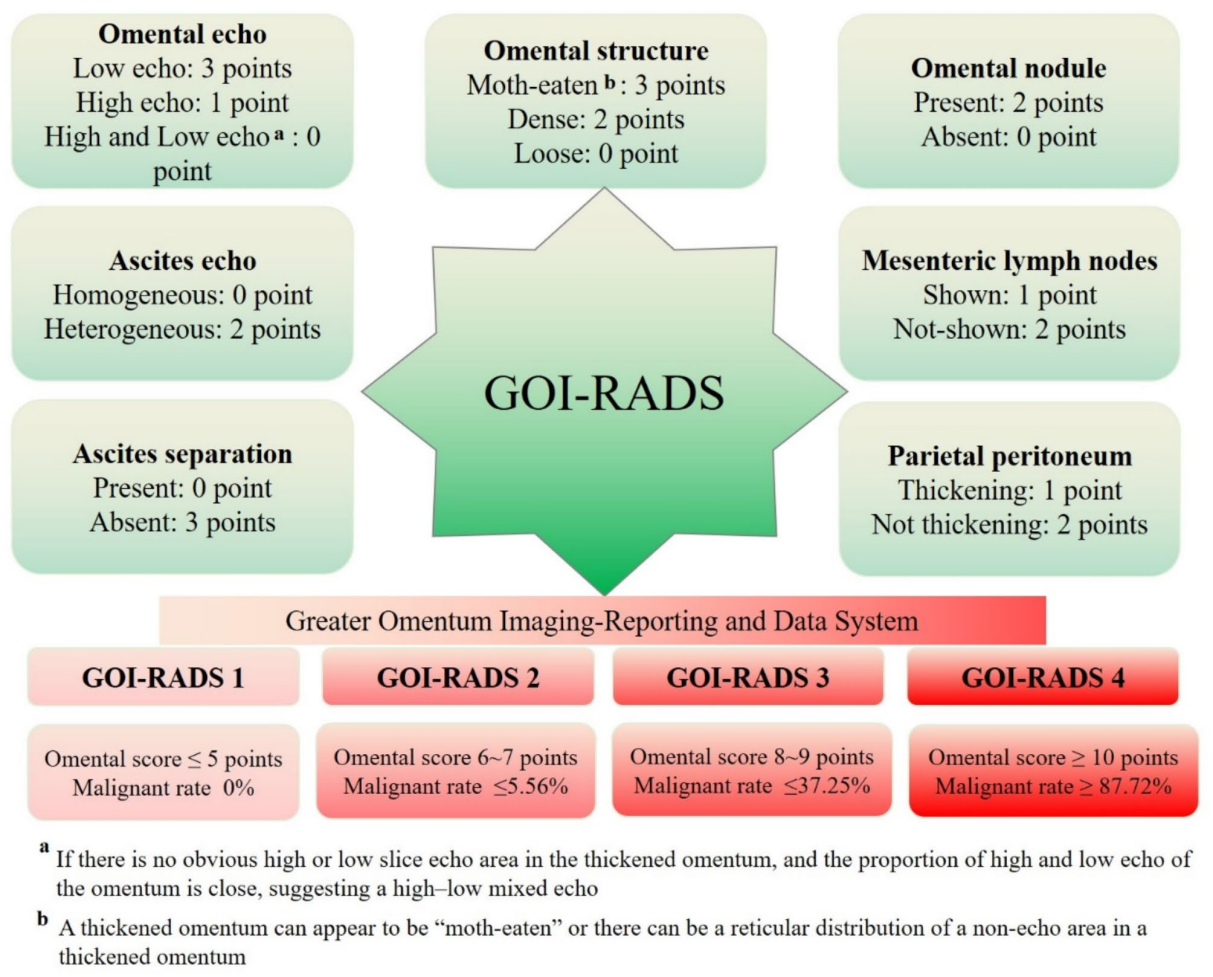
Scoring strategy of GOI-RADS

A retrospective study was conducted to collect patients who underwent peritoneal ultrasonography in our hospital from November 2019 to November 2021. The peritoneal lesions were classified according to the omental scoring (Fig. 2). After obtaining the omental pathology through ultrasound-guided puncture biopsy, the concordance rate and accuracy of the GOI-RADS classification were validated (Fig. 1A). The omental scoring scheme is as follows [12]:

Direct signs of omental lesions:

(i) Omental echo: 3 points for low echo, 1 point for high echo, 0 points for mixed high and low echo.
(ii) Omental structure: 3 points for moth-eaten appearance, 2 points for dense structure, 0 points for loose structure.
(iii) Omental nodule: 2 points if present, 0 points if absent.

Indirect signs of omental lesions:

(i) Ascites separation: 0 points if present, 3 points if absent.
(ii) Ascites echo: 0 points for homogeneous echo, 2 points for heterogeneous echo.
(iii) Mesenteric lymph nodes: 1 point if shown, 2 points if not shown (indicating absence or normal appearance).
(iv) Parietal peritoneum: 1 point for thickening, 2 points for no thickening (indicating normal or minimal thickening).

### Developing a novel classification system based on CEUS and RTE findings

Our team’s research findings have demonstrated that ultrasound elastography can serve as a non-invasive imaging modality for differentiating benign from malignant omental lesions [9]. Specifically, when the elasticity score is 3 or 4, ultrasound elastography indicates a malignant diagnosis for omental lesions, whereas a score of 1 or 2 suggests a benign diagnosis. During CEUS examinations for omental lesions conducted by our team, we observed that malignant omental lesions often exhibit rapid radial enhancement patterns [10]. Therefore, when CEUS reveals rapid enhancement in omental lesions, it is indicative of a malignant diagnosis; conversely, iso-enhancement or slow enhancement patterns suggest a benign diagnosis. Based on our research [8], the grading system considers omental lesions as benign when they are classified as GOI-RADS 1–3, and as malignant when categorized as GOI-RADS 4.

#### Combined diagnosis

Combined Diagnosis of RTE and GOI-RADS (RTE + GOI-RADS): When both RTE and GOI-RADS classify the omental lesion as benign, the RTE + GOI-RADS diagnosis is benign; otherwise, it is malignant.

Combined Diagnosis of CEUS and GOI-RADS (CEUS + GOI-RADS): When both CEUS and GOI-RADS deem the omental lesion as benign, the CEUS + GOI-RADS diagnosis is benign; otherwise, it is malignant.

**Fig. 3:**
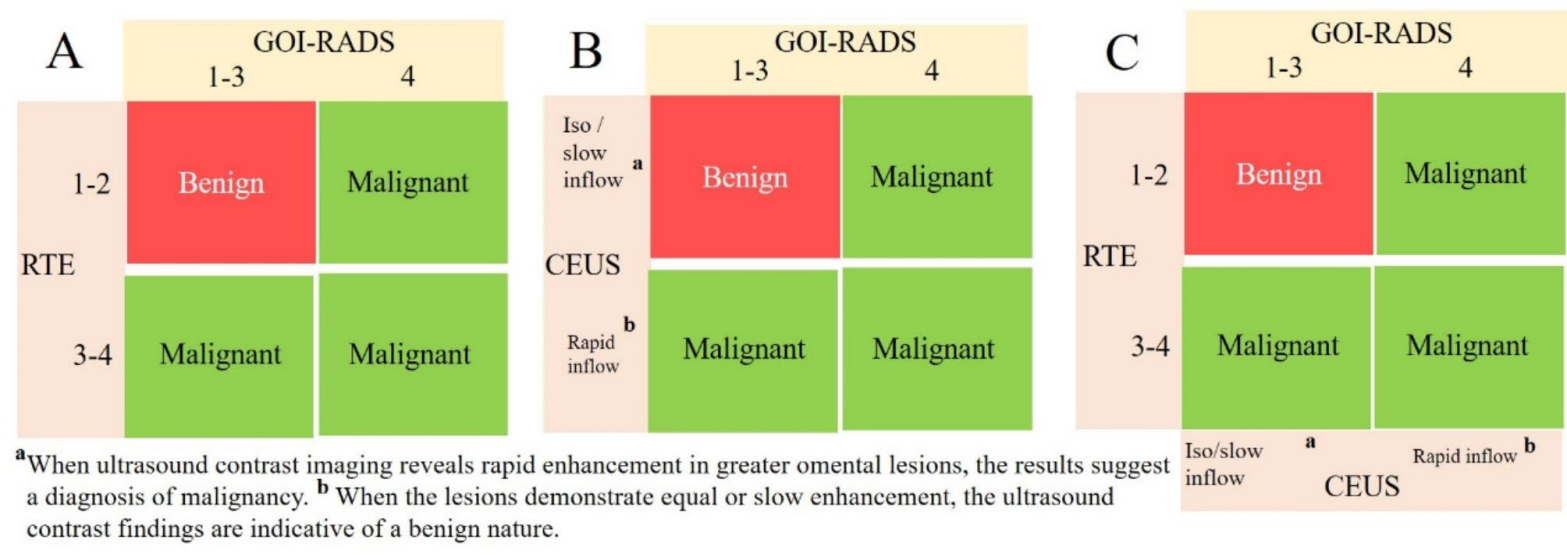
Combined diagnostic strategies. (**A**) Integrated diagnosis combining GOI-RADS and RTE; (**B**) Integrated diagnosis combining GOI-RADS and CEUS; (**C**) Comprehensive integrated diagnosis combining GOI-RADS, RTE, and CEUS

Combined Diagnosis of RTE, CEUS, and GOI-RADS (CEUS + RTE + GOI-RADS): When all three - RTE, CEUS, and GOI-RADS - concur that the omental lesion is benign, the CEUS + RTE + GOI-RADS diagnosis is benign; otherwise, it is malignant (Fig. 3).

#### Building a new grading system

A retrospective analysis was conducted to collect data from patients with omental lesions who underwent pathological examinations between November 2019 and November 2021 (inclusion and exclusion criteria are detailed in Fig. 1B). The results of ultrasound elastography (elasticity scores: 1 point, 2 points, 3 points, and 4 points) and CEUS findings were documented. CEUS findings encompassed the enhancement pattern of the omentum (homogeneous vs. heterogeneous enhancement), the time of contrast agent entry into the omental tissue, time to peak enhancement, and washout time. Additionally, the pattern of contrast agent entry (fast, iso, or slow) and washout (fast, iso, or slow) were compared with the surrounding tissues.

Using pathological outcomes as the gold standard, patients with omental lesions were categorized into benign and malignant groups. The odds ratio (OR) values for CEUS and RTE results were calculated separately for both groups. Based on these OR values, scores were assigned to CEUS and elastography results, which were then incorporated into the GOI-RADS scoring system to develop a novel grading system. Following our previous research, OR values of < 0.3, 0.3 ≤ OR < 3, 3 ≤ OR < 10, and OR ≥ 10 were assigned 0, 1, 2, and 3 points, respectively [8].

### Simplify the new grading system

Retrospectively, data on patients with pathological outcomes of omental diseases from November 2019 to November 2021 were collected, and their conventional ultrasound signs (as described in Sect. 2.3) were statistically analyzed. The results of CEUS and RTE followed the same methodology outlined in Sect. 2.4.2. Drawing upon our previous research experience in simplifying the American College of Radiology Thyroid Imaging Reporting and Data System [11], we assigned a score of 0 to ultrasound signs with an OR < 1 and a score of 1 to those with an OR > 1. This approach facilitated the simplification of the scoring process for the new grading system.

### Prospective validation of the new grading system and its simplified version

Prospectively, patients with peritoneal lesions who consecutively visited our hospital from November 2021 to June 2024 were enrolled, with the relevant inclusion and exclusion criteria detailed in Fig. 1D. The patients’ conventional ultrasound signs, RET, and CEUS results were collected, following the same methodology outlined in Sect. 2.5. Subsequently, all examination results, with CEUS results in AVI format, were reviewed separately by three sonographers with varying levels of experience (WXM with 30 years of work experience, CZG with 5 years, and BZQ with 1 year). The diagnostic consistency among the three sonographers was assessed, and the time taken for each physician’s review, the accuracy of omental scoring, and the classification of omental lesions were statistically analyzed to identify the most clinically suitable classification system. The Intraclass Correlation Coefficient (ICC) results were interpreted as follows: ICC < 0.50 indicate poor reliability, 0.50 ≤ ICC< 0.75 indicate moderate reliability, 0.75 ≤ ICC< 0.90 indicate good reliability, and ICC ≥ 0.90 indicate excellent reliability.

### Statistical analysis

Statistical analyses were performed using SPSS Statistics 25.0 (IBM Corporation, New York, USA). Categorical data were presented as frequencies or percentages, and continuous data were expressed as mean ± standard deviation. To evaluate the diagnostic value of CEUS parameters, RTE, and GOI-RADS, either alone or in combination, for benign and malignant omental diseases, the Chi-square test was employed. For continuous variables such as patient age and omental thickness, an independent samples t-test was used. The OR between the benign and malignant groups was calculated using the formula (True positive/False negative) / (False positive/True negative), and corresponding scores were assigned. Receiver Operating Characteristic (ROC) curves were constructed to analyze the diagnostic performance of the newly grading system for malignant omental lesions. Inter-rater and intra-rater agreement were assessed using the ICC. *P* < 0.05 was considered statistically significant.

## Results

### Clinical application results of GOI-RADS

From November 2019 to November 2021, a total of 624 patients underwent ultrasound examinations at our hospital with suspected peritoneal lesions. Among them, 224 patients underwent ultrasound-guided greater omentum biopsy, and 215 patients had definitive pathological results (49 benign and 166 malignant cases) (Fig. 1A). The 215 patients ranged in age from 20 to 90 years, with a mean age of 58.59 ± 12.33 years. There were 57 male patients and 158 female patients (Supplementary Table 1).

Based on the GOI-RADS classification, 7 patients were categorized as GOI-RADS 1, 10 as GOI-RADS 2, 49 as GOI-RADS 3, and 149 as GOI-RADS 4. A comparison of the actual malignancy rate with the predicted malignancy rate for each category is presented in Table 1.

**Table 1:** Clinical application results of GOI-RADS

	Pathological results		Actual malignancy (%)	Predicted malignancy (%)
	Benign (*n* = 49)	Malignant (*n* = 166)		
GOI-RADS 1	7	0	0	0
GOI-RADS 2	6	4	40.00	≤ 5.56
GOI-RADS 3	19	30	61.22	≤ 37.25
GOI-RADS 4	15	134	89.92	>87.72

The actual malignancy rates for GOI-RADS 1 and GOI-RADS 4 were in good agreement with their predicted malignancy rates, whereas the actual malignancy rates for GOI-RADS 2 and GOI-RADS 3 were significantly higher than their predicted rates, resulting in a diagnostic accuracy of only 77.21% (166/215). Upon re-analysis of the data, we identified a common factor contributing to the elevated actual malignancy rates in GOI-RADS 2 and GOI-RADS 3 categories. This factor was observed in a subset of patients with omental lesions that manifested solely as hyperechoic and densely structured, without other abnormal ultrasonic signs. Consequently, these lesions scored between 6 and 10 points on the omental scoring system. In clinical practice, we have found that omental lesions with such echotexture and structure can be seen in both metastatic cancers and tuberculosis, necessitating the use of additional imaging modalities for diagnostic support.

### Developing a new classification system based on CEUS and RTE results

To enhance the diagnostic accuracy and agreement rates for GOI-RADS 2 and GOI-RADS 3, we incorporated ultrasound elastography [13] and CEUS [14] into the GOI-RADS framework to further differentiate between benign and malignant omental lesions. Among the 215 patients, 104 underwent both RTE and CEUS, including 18 cases of benign lesions, 86 cases of malignant lesions, 22 males, and 82 females, ranging in age from 24 to 87 years, with a mean age of 58.81 ± 12.80 years (Supplementary Table 2).

**Table 2:** Diagnostic efficacy of CEUS, RTE, and GOI-RADS alone and in combination

		Pathological results		Sen(%)	Spe(%)	Accuracy(%)	PPV(%)	NPV(%)
		Benign (n=18)	Malignancy (n=86)					
Elastic score				81.4	66.67	78.85	92.11	42.86
	1–2	12	16					
	3–4	6	70					
CEUS				91.86	61.11	86.54	91.86	61.11
	Benign	11	7					
	Malignancy	7	79					
GOI-RADS				81.4	61.11	77.88	90.91	40.74
	1–3	11	16					
	4	7	70					
RTE + CEUS								
	Benign	8	0	100	44.44	90.38	89.58	100
	Malignancy	10	86					
RTE + GOI-RADS								
	Benign	10	4	95.35	55.56	88.46	91.11	71.43
	Malignancy	8	82					
CEUS + GOI-RADS								
	Benign	8	3	96.51	44.44	87.50	89.25	72.73
	Malignancy	10	83					
RTE + CEUS + GOI-RADS								
	Benign	7	0	100	38.89	89.42	88.67	100
	Malignancy	11	86					

Firstly, we evaluated the diagnostic performance of RTE and CEUS individually for benign and malignant omental lesions (Table 2). CEUS, RTE, and GOI-RADS all demonstrated high positive predictive values (PPV) (all > 90%), with CEUS exhibiting the highest sensitivity (91.86%) and accuracy (86.54%). However, all three diagnostic methods had relatively low specificity when used individually (all < 70%).

To further enhance diagnostic sensitivity and accuracy, we employed various combinations of RTE, CEUS, and GOI-RADS for joint diagnosis (Table 2). The combination of RTE and CEUS was designated RTE + CEUS, RTE with GOI-RADS as RTE + GOI-RADS, CEUS with GOI-RADS as CEUS + GOI-RADS, and the integration of RTE, CEUS, and GOI-RADS as CEUS + RTE + GOI-RADS. We found that combined application significantly improved diagnostic sensitivity and accuracy, particularly RTE + CEUS and CEUS + RTE + GOI-RADS, which achieved 100% sensitivity and 90.38% and 89.42% accuracy, respectively. Among these combined diagnostic approaches, RTE + GOI-RADS exhibited the highest area under the curve (AUC) (0.76, 95%CI: 0.61, 0.90), as shown in Supplementary Fig. 1. However, combined use further decreased diagnostic specificity, with CEUS + RTE + GOI-RADS showing the lowest specificity at 38.89%.

**Table 3:** Elastic score and CEUS results of 104 patients

		Pathological results		t/χ^2^	*P*	OR	Assignment
		Malignancy (n=86)	Benign (n=18)				
Elasticity score							
	1–2 points	16	12	17.48	0.001	0.11	0
	3–4 points	70	6			8.75	2
CEUS							
Enhancement patterns	homogeneous	13	10	14.13	0.001	0.14	0
	heterogeneous	73	8			7.02	2
Contrast agent entry							
	Rapid	79	7	24.72	0.001*	17.73	3
	Iso	7	9			0.06	0
	slow	0	2			NA	0
Contrast agent washout							
	Rapid	42	5	3.25	0.208*	NA	NA
	Iso	41	13				
	slow	3	0				
Times						NA	NA
Time to onset of enhancement		10.69 ± 4.23	10.89 ± 4.23	0.48	0.833		
Time to peak enhancement		19.83 ± 5.74	17.56 ± 5.66	0.003	0.129		
Time to onset of washout		35.7 ± 23.93	45.89 ± 19.85	1.38	0.095		

* Fisher’s exact test; Sen: sensitivity; Spe: specificity; PPV: positive predictive value; NPV: negative predictive value

To increase diagnostic specificity, we propose incorporating elasticity scores and CEUS parameters into the GOI-RADS scoring system. Table 3 presents the distribution of elasticity scores and CEUS parameters among the 104 patients. Statistically significant differences were observed in elasticity scores, CEUS enhancement patterns, and contrast agent entry patterns between the benign and malignant omental lesion groups (all *P* < 0.001). Based on calculated OR values, we assigned scores to corresponding parameters: an elasticity score of 3–4 was assigned 2 points, while 1–2 received 0 points; heterogeneous enhancement on CEUS was assigned 2 points and homogeneous enhancement 0 points; rapid contrast agent entry was assigned 3 points, and iso- or slow entry was assigned 0 points.

**Fig. 4:**
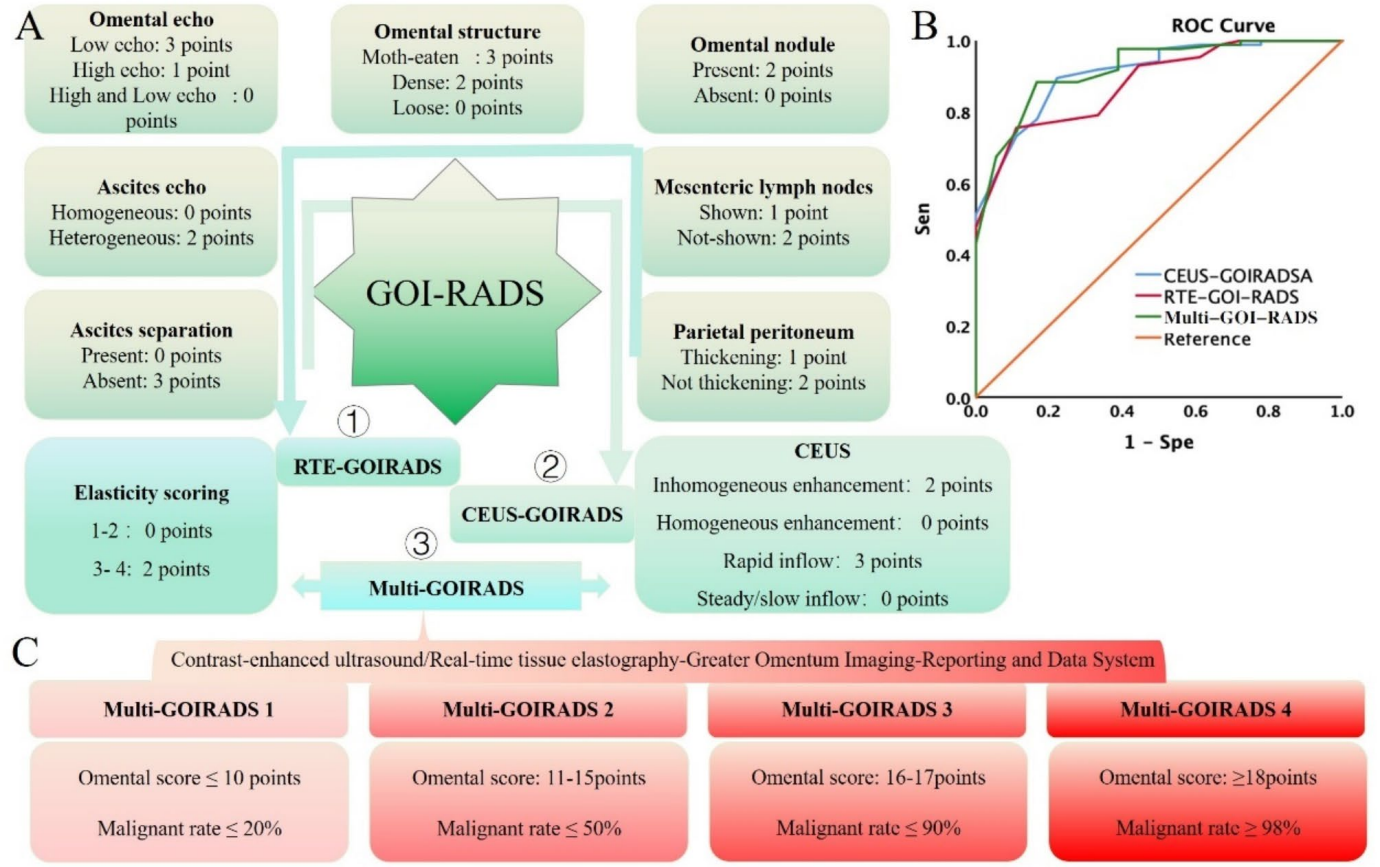
Developing a new grading system based on CEUS and RTE. (**A**) Scoring strategies for the new grading system: ① RTE-GIORADS: Constructed solely based on RTE results; ②CEUS-GOIRADS: Constructed solely based on CEUS findings; ③Multi-GOIRADS: Integratively constructed by incorporating both CEUS and RTE results. (**B**) AUC for the three grading systems: AUC of RTE-GIORADS: 0.88 (95% CI: 0.81, 0.95), AUC of CEUS-GOIRADS: 0.91 (95% CI: 0.84, 0.98), AUC of Multi-GOIRADS: 0.92 (95% CI: 0.86, 0.98); (**C**) Categorization of Multi-GOIRADS into four categories with corresponding omental scores and malignancy rates

We have incorporated elasticity scores and CEUS parameters into the GOI-RADS assessment process, creating a novel grading system. When only elasticity scores are included in GOI-RADS, it is designated as RTE-GOIRADS. When only CEUS parameters are included, it is designated as CEUS-GOIRADS. When both elasticity scores and CEUS parameters are simultaneously incorporated into GOI-RADS, it is designated as Multi-GOIRADS (Fig. 4A).

By constructing an ROC curve, we found that Multi-GOIRADS had the largest AUC of 0.92 (95% CI: 0.86, 0.98) (Fig. 4B). When a cutoff value of ≥ 15.5 for the omental score was used to diagnose malignant omental lesions, the diagnostic sensitivity, specificity, accuracy, positive predictive value (PPV), and negative predictive value (NPV) were 88.37%, 83.33%, 87.50%, 96.20%, and 60.00%, respectively (Table 4).

**Table 4:** Diagnostic efficacy of the new grading system

	AUC	95%CI		Sen(%)	Spe(%)	Accuracy(%)	PPV(%)	NPV(%)	Youden’s index
		Lower	upper						
RTE-GOIRADS	0.88	0.81	0.95	75.58	88.89	77.88	97.01	43.24	0.6447
CEUS-GOIRADS	0.91	0.84	0.98	89.53	77.78	87.50	95.06	60.87	0.6731
Multi -GOIRADS	0.92	0.86	0.98	88.37	83.33	87.50	96.20	60.00	0.7170

Interestingly, incorporating elasticity scores and CEUS parameters into the GOI-RADS scoring system not only significantly enhances diagnostic specificity but also further improves the AUC. Consequently, we calculated the malignancy rates corresponding to the omental scores of Multi-GOIRADS **(Supplementary Table 3)**. Based on these malignancy rates, we classified Multi-GOIRADS into four categories (Fig. 4C): Multi-GOIRADS 1, with an omental score ≤ 10 points and a malignancy rate ≤ 20%; Multi-GOIRADS 2, with an omental score of 11–15 points and a malignancy rate of 20–50%; Multi-GOIRADS 3, with an omental score of 16–17 points and a malignancy rate of 50–90%; and Multi-GOIRADS 4, with an omental score ≥ 18 points and a malignancy rate ≥ 98%. This system enriches the comprehensiveness of omental scoring, particularly in terms of the diversity of scoring parameters, but also introduces complexity to the omental scoring system.

### Simplify the multi-GOIRADS

**Fig. 5:**
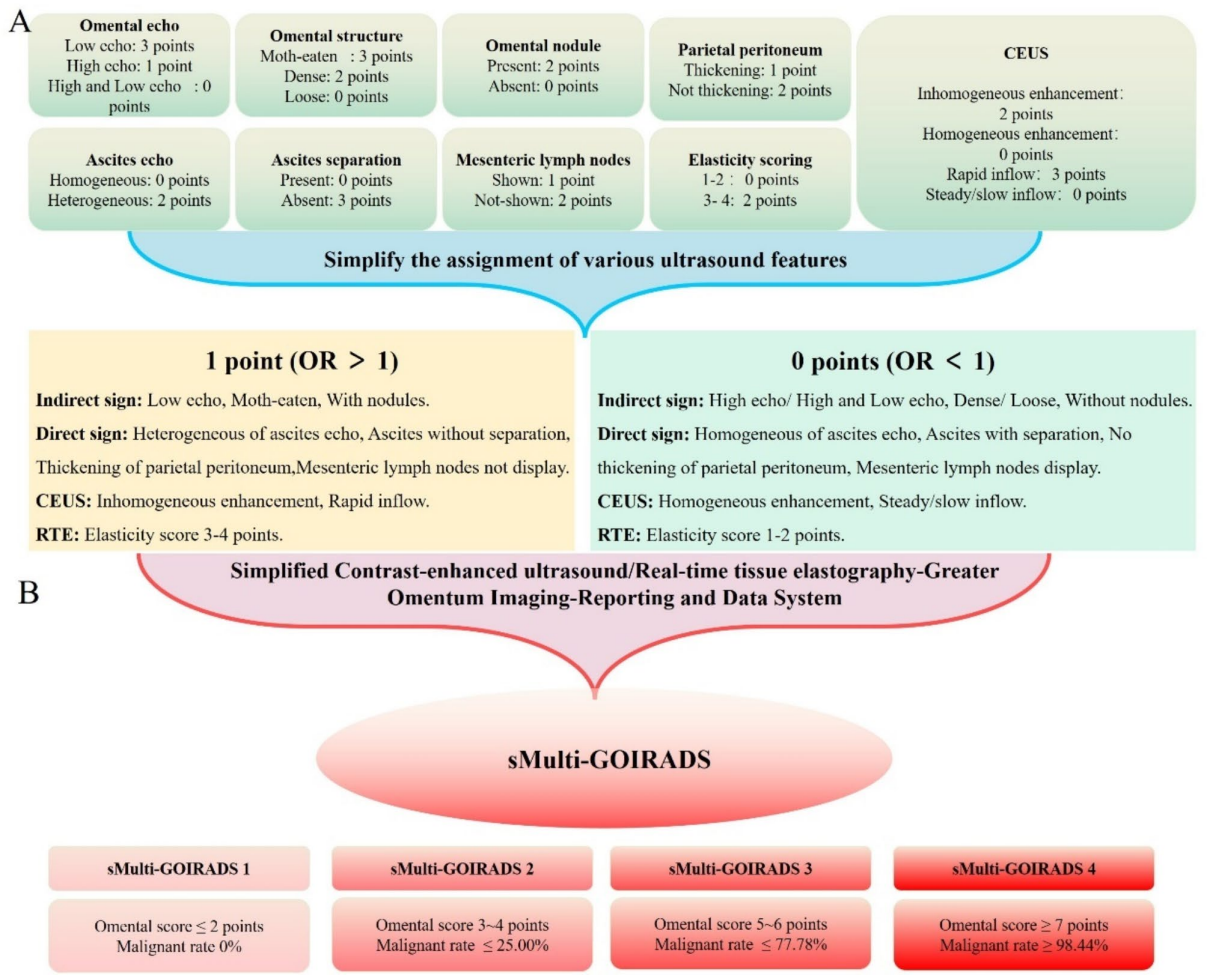
Simplified the scoring process and establishment of the simplified Multi-GOIRADS. (**A**) Simplification of the Multi-GOIRADS Scoring Process: To simplify the process, we assigned a score of 1 to variables with OR > 1 and a score of 0 to those with OR < 1. (**B**) Establish sMulti-GOIRADS and classify into four categories, each category corresponds to a specific omental score and malignancy

To streamline the complex omental scoring system of Multi-GOIRADS, we propose simplifying the scoring process. By reassessing and reanalyzing the conventional ultrasonic signs, CEUS, and elasticity scores results of omental lesions, we have compiled Table 5. By calculating the OR of each parameter for benign and malignant omental lesions, we assigned 1 point to the following ultrasonic signs or parameters: hypoechoic, wormhole/grid-like pattern, with nodules, no thickening of parietal peritoneum, no enlargement of mesenteric lymph nodes, heterogeneous of ascites echo, no ascites septation, elasticity score of 3–4, heterogeneous enhancement, and rapid contrast agent entry. All other ultrasonic signs or parameters were assigned 0 points (Fig. 5A).

**Table 5:** Routine ultrasound signs, CEUS, and RTE results of omental lesions

Ultrasonic signs		Malignancy (n=166)	Benign (n=49)	OR	Assignment
Omental echo					
	hyperechoic	92	38	0.36	0
	hypoechoic	69	2	16.72	1
	High-low mixed echo	5	9	0.14	0
Omental structure					
	loose	7	18	0.08	0
	dense	74	28	0.60	0
	Moth-eaten	85	3	16.09	1
Nodules					
	with	76	10	3.29	1
	without	90	39	0.30	0
Peritoneum wall					
	Thickening	20	16	0.28	0
	Not thickening	146	33	3.54	1
Mesenteric lymph node					
	Display	4	7	0.15	0
	Not display	162	42	6.75	1
Ascites echo					
	Homogeneous	97	42	0.23	0
	heterogeneous	69	7	4.27	1
Ascites separation					
	With	5	9	0.14	0
	without	161	40	7.25	1
		Malignancy (n=86)	Benign (n=18)		
Elasticity score					
	1–2 points	16	12	0.11	0
	3–4 points	70	6	8.75	1
Enhancement patterns					
	homogeneous	13	10	0.14	0
	heterogeneous	73	8	7.02	1
Contrast agent entry					
	Rapid	79	7	17.73	1
	Iso	7	9	0.06	0
	slow	0	2	NA	0

Subsequently, we simplified the scoring of ultrasonic signs or parameters from 104 patients with omental lesions. By establishing a ROC curve, we obtained an AUC of 0.92 (95% CI: 0.85, 0.98) for the simplified Multi-GOIRADS (sMulti-GOIRADS) **(Supplementary Fig. 2)**. When using an omental score ≥ 6.5 points as the cut-off for diagnosing malignant omental lesions, the diagnostic sensitivity, specificity, accuracy, PPV, and NPV were 73.26%, 94.44%, 76.92%, 98.43%, and 42.50%, respectively **(Supplementary Table 4).**

We then calculated the malignancy rates corresponding to the omental scores of sMulti-GOIRADS (Supplementary Table 5). Based on these malignancy rates, we categorized sMulti-GOIRADS into four classes (Fig. 5B): sMulti-GOIRADS 1, with an omental score ≤ 2 points and a malignancy rate of 0%; sMulti-GOIRADS 2, with an omental score of 3–4 points and a malignancy rate ≤ 25%; sMulti-GOIRADS 3, with an omental score of 5–6 points and a malignancy rate ≤ 77.78%; and sMulti-GOIRADS 4, with an omental score ≥ 7 points and a malignancy rate ≥ 98.44%.

Compared to Multi-GOIRADS, sMulti-GOIRADS reduces the malignancy rates associated with categories 1–3, effectively classifying nodules with suspected malignancy into higher categories. However, prospective studies are necessary to evaluate the clinical practicability and portability of the proposed GOI-RADS, Multi-GOIRADS, and sMulti-GOIRADS systems.

**Fig. 6:**
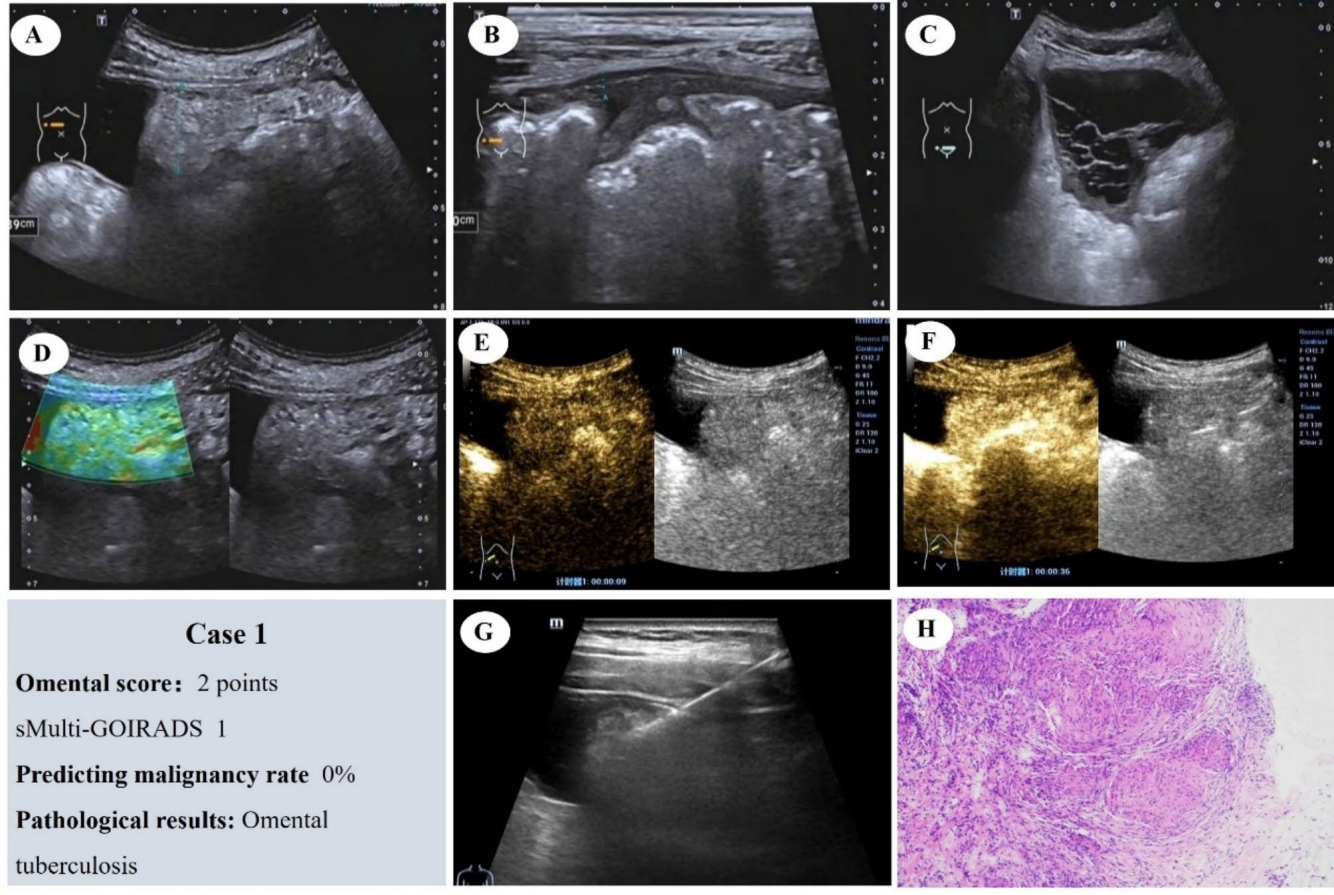
Omental Score: 2 Points, sMulti-GOIRADS 1, predicting Benign Omental Lesion. (**A**) The omentum appears hyperechoic, dense and without nodules. (**B**) Thickening of the parietal peritoneum. (**C**) Ascites with separation, with homogeneous echo. Mesenteric lymph node not display. (**D**) Elastography score, 1 point. (**E**) CEUS reveals iso-enhancement. (**F**) CEUS show heterogeneous enhancement. (**G**) Ultrasound-guided aspiration biopsy of the omentum is performed. (**H**) Pathological examination reveals granulomatous lesions with chronic inflammation (HE staining), confirming the benign nature of the omental lesion

**Fig. 7:**
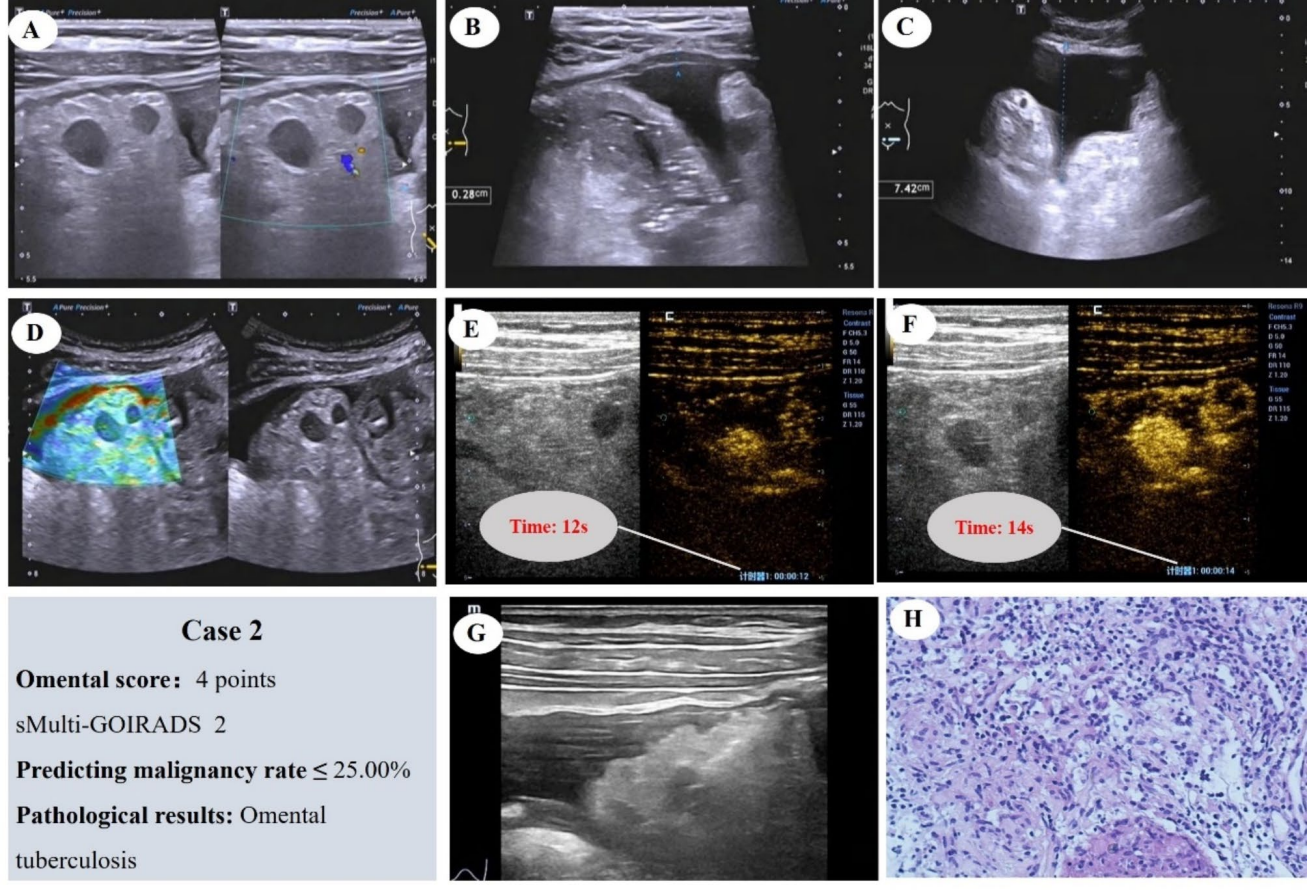
Omental Score: 4 Points, sMulti-GOIRADS 2, predicting the nature of omental lesions as benign is highly likely. (**A**) The omentum appears hyperechoic, dense and with nodules. (**B**) Thickening of the parietal peritoneum. (**C**) Ascites without separation, with homogeneous echo. Mesenteric lymph node not display. (**D**) Elastography score, 2 points. (**E**) CEUS reveals rapid-enhancement. (**F**) CEUS show heterogeneous enhancement. (**G**) Ultrasound-guided aspiration biopsy of the omentum is performed. (**H**) Pathological examination reveals chronic granulomatous lesions (HE staining)

**Fig. 8:**
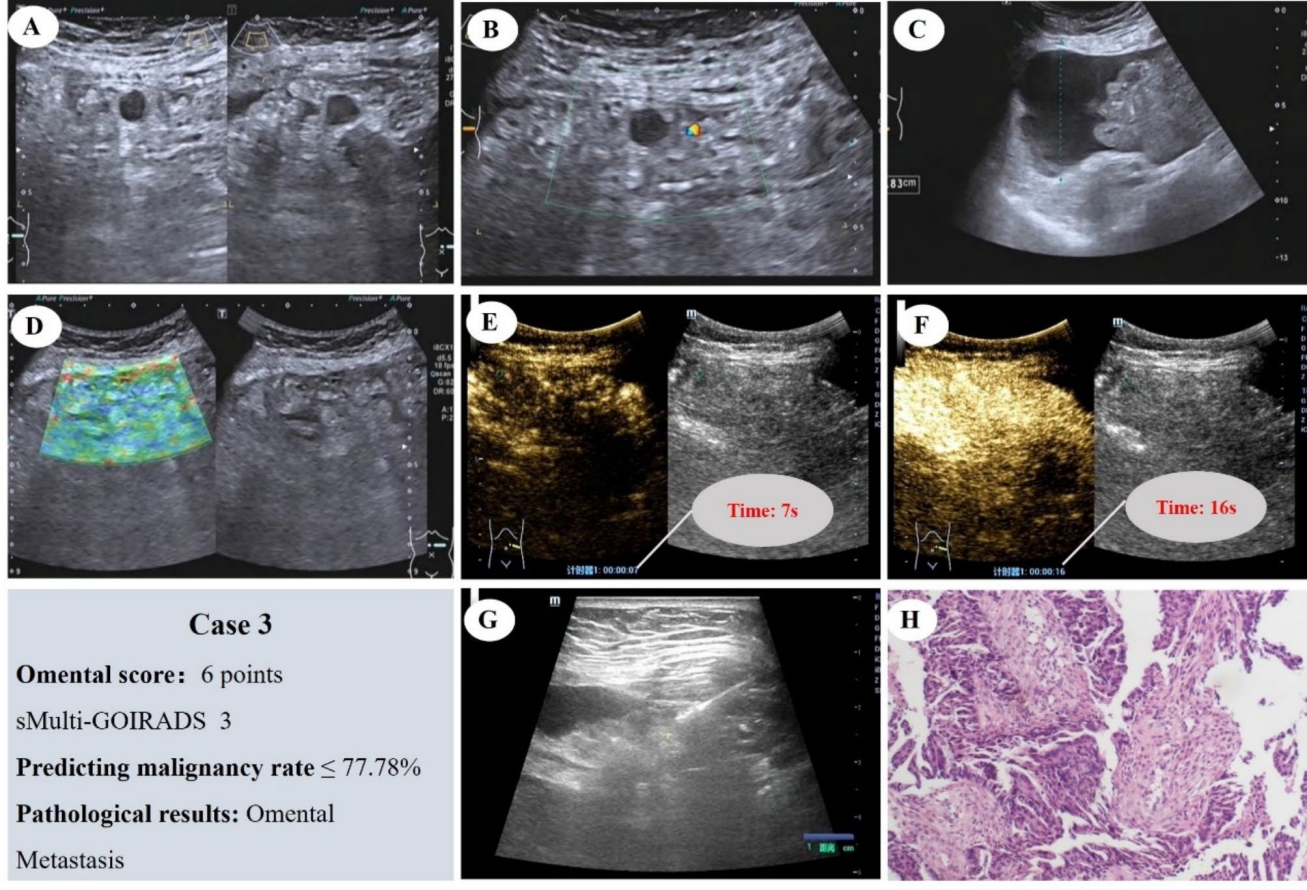
Omental Score: 6 Points, sMulti-GOIRADS 3, predicting omental lesions as having a high likelihood of malignancy. (**A**) The omentum appears hyperechoic, dense and with nodules. (**B**) Not thickening of the parietal peritoneum. (**C**) Ascites without separation, with homogeneous echo. Mesenteric lymph node not display. (**D**) Elastography score, 3 points. (**E**) CEUS reveals rapid-enhancement. (**F**) CEUS show heterogeneous enhancement. (**G**) Ultrasound-guided aspiration biopsy of the omentum is performed. (**H**) Pathological examination reveals metastatic cancer, likely of ovarian origin (HE staining)

**Fig. 9:**
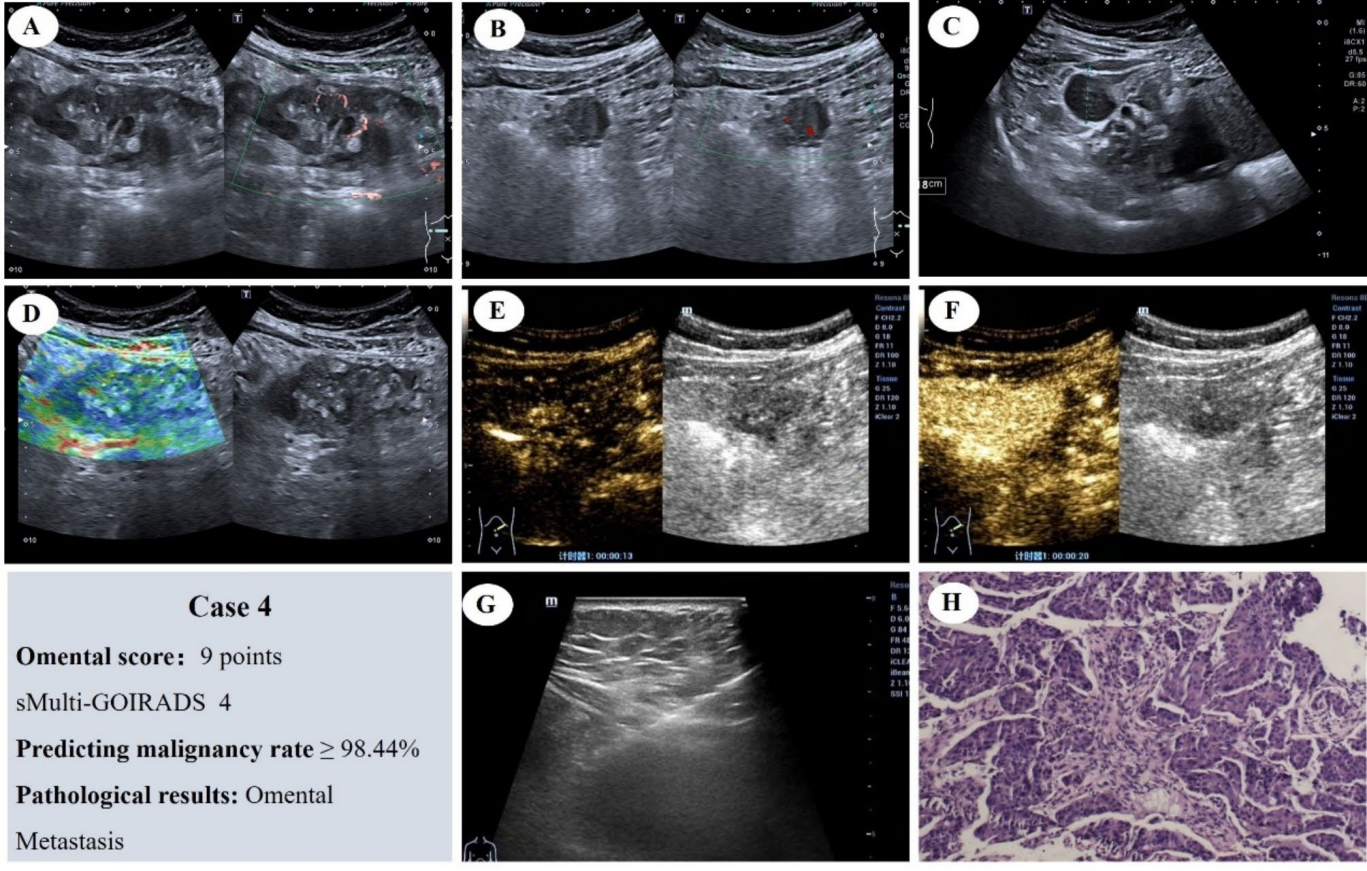
Omental Score: 9 Points, sMulti-GOIRADS 4, predicting omental lesions as malignant. (**A**) The omentum appears hypoechoic, moth-eaten. (**B**) With nodules, not thickening of the parietal peritoneum. (**C**) Ascites without separation, with homogeneous echo. Mesenteric lymph node not display. (**D**) Elastography score, 3 points. (**E**) CEUS reveals rapid-enhancement. (**F**) CEUS show heterogeneous enhancement. (**G**) Ultrasound-guided aspiration biopsy of the omentum is performed. (**H**) Pathological examination reveals metastatic carcinoma (adenocarcinoma) (HE staining)

Figures 6, 7, 8 and 9 illustrate clinical cases belonging to sMulti-GOIRADS categories 1 through 4, respectively. Figure 6 depicts a case with an omental score of 2, classified as sMulti-GOIRADS 1. The ultrasound prediction indicates a benign nature of the lesion, which is subsequently confirmed by pathology as a granulomatous lesion with chronic inflammation.

Fig. 7 shows a case with an omental score of 4, classified as sMulti-GOIRADS 2. The ultrasound prediction suggests a high likelihood of benignity, which is subsequently confirmed by pathology as a chronic granulomatous lesion

Fig. 8 depicts a case with an omental score of 6, classified as sMulti-GOIRADS 3. The ultrasound prediction indicates a high probability of malignancy, which is then confirmed by pathology as metastatic carcinoma, likely of ovarian origin

Fig. 9 displays a case with an omental score of 9, classified as sMulti-GOIRADS 4. The ultrasound prediction asserts the malignancy of the lesion, which is indeed verified by pathology as metastatic carcinoma (adenocarcinoma)

### Prospective application validation

Prospective data were collected from 1123 consecutive patients with peritoneal lesions who presented from November 2021 to June 2024. Among these, 356 underwent ultrasound-guided greater omentum biopsy, and only 138 patients had complete data for ultrasound elastography, CEUS, and conventional ultrasound (Fig. 1D). Three sonographers with varying levels of expertise categorized the 138 patients using both sMulti-GOIRADS and Multi-GOIRADS. Excellent inter-rater agreement was observed among the three sonographers when scoring omental lesions using both sMulti-GOIRADS (ICC = 0.90) and Multi-GOIRADS (ICC = 0.89). When comparing the physicians in pairs, the use of sMulti-GOIRADS yielded excellent consistency in omental scoring (ICC = 0.92 for WXM and CZG, ICC = 0.91 for WXM and BZQ, ICC = 0.92 for BZQ and CZG), while Multi-GOIRADS demonstrated good consistency (ICC = 0.89 for WXM and CZG, ICC = 0.84 for WXM and BZQ, ICC = 0.89 for BZQ and CZG).

Among the 138 patients, 38 were male and 100 were female, with ages ranging from 29 to 86 years and a mean age of 60.58 ± 10.52 years. When using sMulti-GOIRADS to predict omental lesions, all three sonographers exhibited high specificity (100%, 97.06%, and 100%, respectively) but relatively low sensitivity (68.72%, 60.58%, and 60.58%, respectively). In contrast, when using Multi-GOIRADS, the sensitivity exceeded 80% with high specificity (91.43%, 82.35%, and 91.18%, respectively). Notably, Doctor 1 achieved the highest Youden’s index (0.7978) when predicting benign and malignant omental lesions using Multi-GOIRADS (Table 5).

Interestingly, the time taken by all three sonographers to classify patients using sMulti-GOIRADS was significantly shorter than that using Multi-GOIRADS (*P* < 0.001 for all comparisons) (Table 6).

**Table 6:** Evaluation of benign and malignant lesions in 138 patients by three sonographers using sMulti-GOIRADS and Multi-GOIRADS

	Doctor 1(WXM)		Doctor 2(CZG)		Doctor3(BZQ)	
	sMulti-GOIRADS	Multi-GOIRADS	sMulti-GOIRADS	Multi-GOIRADS	sMulti-GOIRADS	Multi-GOIRADS
Times	59.16 ± 12.90	78.25 ± 24.30	60.14 ± 11.84	92.96 ± 25.78	67.86 ± 12.16	121.59 ± 60.23
t	8.151		13.83		11.76	
*P*	0.001		0.001		0.001	
Sen(%)	68.27	88.35	60.58	81.73	60.58	80.77
Spe(%)	100.00	91.43	97.06	82.35	100.00	91.18
Accuracy(%)	76.09	89.13	69.57	81.88	70.29	83.33
PPV(%)	100.00	96.81	98.44	93.41	100.00	96.55
NPV(%)	50.75	72.73	44.59	59.57	45.33	60.78
Youden’s index	0.6827	0.7978	0.5764	0.6408	0.6058	0.7195

## Discussion

In this study, the application efficacy of GOI-RADS was validated, expanded, optimized, and evaluated. By incorporating CEUS and elasticity score results, a new grading system, named Multi-GOIRADS, was constructed based on multiple ultrasound parameters. This system achieved a sensitivity of 88.37%, a specificity of 83.33%, and an accuracy of 87.50% in diagnosing malignant omental lesions. While enhancing the comprehensiveness of omental scoring, particularly in terms of the diversity of scoring parameters, this system introduced complexity. Therefore, the assignment parameters were simplified to obtain the simplified Multi-GOIRADS, termed sMulti-GOIIRADS, which significantly reduced scoring time and enhanced inter-operator consistency.

Initially, a retrospective analysis was conducted to assess the accuracy and concordance rate of GOI-RADS in differentiating benign from malignant omental lesions. The diagnostic accuracy of GOI-RADS was found to be 77.21% (166/215). The actual and predicted malignancy rates for GOI-RADS 1 and GOI-RADS 4 were relatively consistent, but the actual malignancy rates for GOI-RADS 2 and GOI-RADS 3 were significantly higher than predicted. Reanalysis of the ultrasound images revealed that the high echogenicity and dense structure of the affected omentum, without accompanying abnormal ultrasound signs, primarily contributed to scores ranging from 6 to 9. Studies have suggested that omental tuberculosis commonly exhibits high echogenicity, while peritoneal carcinoma often shows low echogenicity. However, approximately 20% of peritoneal carcinoma cases still present with high echogenicity [12]. Currently, there is no unified standard for interpreting omental echogenicity. Perez et al. [13] used adjacent abdominal wall fat as a reference and found that 64% of omental lesions were hypoechoic, while 36% were isoechoic or hyperechoic. In this study, the rectus abdominis muscle was used as a reference, defining lesions with higher echogenicity than the muscle as hyperechoic and those with lower echogenicity as hypoechoic. Consequently, most lesions (60.47%, 130/215) were hyperechoic, and hyperechoic lesions accounted for 55.42% (92/166) in the malignant group. Normal omental tissue has a loose structure, but inflammation or tumor cell infiltration can lead to the recruitment of immune cells such as monocyte-like dendritic cells and macrophages in the omentum, resulting in tissue aggregation and a dense structure [14, 15]. Consequently, GOI-RADS alone struggles to definitively characterize omental lesions that exhibit high echogenicity and a dense structure, necessitating the integration of additional diagnostic modalities.

From the initial cohort of 215 patients, 104 were selected who underwent both CEUS and RTE. The analysis revealed that both modalities could independently facilitate the differential diagnosis of omentum diseases with high diagnostic sensitivities: 81.40% for CEUS and 91.86% for RTE, respectively. Subsequently, various configurations combining RTE, CEUS, and the GOI-RADS were explored. It was discovered that the integration of GOI-RADS further enhanced diagnostic sensitivity, particularly when all three methods were combined, achieving a sensitivity of 100% but with a specificity of only 38.89%. This outcome is inherent to parallel testing in combined diagnoses [16]. To address this, the existing framework of GOI-RADS was expanded by incorporating omental elasticity (scores 3–4 assigned 2 points) and CEUS parameters (heterogeneous enhancement assigned 2 points; rapid contrast agent entry assigned 3 points). Based on this integration, three novel grading systems were developed: RTE-GOIRADS, CEUS-GOIRADS, and Multi-GOIRADS. Among these, Multi-GOIRADS exhibited the largest AUC and optimal diagnostic performance when a cut-off score of ≥ 15.5 was used to diagnose malignant greater omentum lesions.

Incorporating omental elasticity results and CEUS parameters into the GOI-RADS scoring system differs from multi-modal ultrasound diagnosis, which combines multiple imaging techniques. Here, the integration of parameters into the omental score establishes a multi-parametric ultrasound grading system that differentiates benign from malignant lesions based on multiple ultrasound parameters. In contrast, multi-modal ultrasound increases sensitivity by increasing the number of positive detections but sacrifices specificity by reducing the number of negative detections. For example, Borlea et al. [17] improved the accuracy and specificity of TI-RADS for thyroid diagnosis by integrating CEUS parameters, while Ruan et al. [18] established the CEUS-TIRADS, a risk stratification system for thyroid nodules incorporating CEUS parameters into TI-RADS scoring, achieving a superior AUC. The inclusion of multiple parameters enhances the comprehensiveness of the omental score but also complicates the scoring process.

To simplify the complexity of Multi-GOIRADS, the omental scoring parameters were optimized. Specifically, ultrasound parameters with an OR greater than 1 were assigned 1 point, while those with an OR less than 1 were assigned 0 points. This led to the development of a new scoring system. By calculating the malignancy rate corresponding to the new omental scores, a simplified version of Multi-GOIRADS was proposed, termed sMulti-GOIRADS. In sMulti-GOIRADS, ultrasound parameters assigned 1 point are categorized into three groups:

1. Direct ultrasound signs: (i) Hypoechoic; (ii) Moth-eaten/reticular pattern; (iii) With nodules.
2. Indirect ultrasound signs: iv) Heterogeneous of ascites echo; v) Ascites without septation; vi) Parietal peritoneum not thickening; vii) Mesenteric lymph node not display.
3. Assisted ultrasound examination: viii) Elasticity score, 3–4 points; ix) Heterogeneous enhancement on CEUS; x) Rapid contrast agent entry.

The AUC for diagnosing benign and malignant omental lesions using sMulti-GOIRADS was 0.92. With an omental score threshold of ≥ 6.5 points, the diagnostic sensitivity, specificity, and accuracy were 73.26%, 94.44%, and 76.92%, respectively.

sMulti-GOIRADS is classified into four categories:

1. sMulti-GOIRADS 1: omental score ≤ 2 points, with a malignancy rate of 0%. In clinical practice, patients with sMulti-GOIRADS 1 should be considered to have benign omental lesions, and tuberculosis antibody testing can be performed to confirm or rule out tuberculous infection, thereby avoiding invasive biopsy.
2. sMulti-GOIRADS 2: omental score 3–4 points, with a malignancy rate ≤ 25.00%. In clinical practice, patients with sMulti-GOIRADS 2 are likely to have benign omental lesions. In the absence of a history of malignancy, tuberculosis antibody testing can be performed to confirm or exclude tuberculous infection.
3. sMulti-GOIRADS 3: omental score 5–6 points, with a malignancy rate ≤ 77.78%. In clinical practice, patients with sMulti-GOIRADS 3 are highly suspected of having malignant omental lesions. When feasible, ultrasound-guided biopsy of the greater omentum should be performed promptly to obtain pathological confirmation of the metastatic source or to undergo PET/CT to identify the primary tumor.
4. sMulti-GOIRADS 4: omental score ≥ 7 points, with a malignancy rate ≥ 98.44%. In clinical practice, patients with sMulti-GOIRADS 4 can be considered to have malignant omental lesions. PET/CT can be performed to identify the primary tumor, or ultrasound-guided biopsy can be done to confirm the metastatic source.

Ultimately, the clinical practicability of sMulti-GOIRADS and Multi-GOIRADS was validated through a prospective study. Three sonographers, possessing varying levels of expertise, exhibited good inter-rater agreement (ICC > 0.80) in assessing omental lesions using both classification systems. Notably, exceptional consistency (ICC > 0.90) was observed specifically when employing sMulti-GOIRADS. The time taken to classify omental lesions using sMulti-GOIRADS was significantly shorter than that required for Multi-GOIRADS. This disparity can be attributed to the relatively complex scoring calculation process and the broader range of omental scores in the Multi-GOIRADS classification system, which may lead to score accumulation errors, thereby affecting inter-rater agreement. When differentiating benign from malignant omental lesions using sMulti-GOIRADS, high specificity was achieved by all three sonographers (100% for WXM and BZQ), but sensitivity (both < 70%) and accuracy (76.09%, 69.57%, and 70.29%, respectively) were lower. In contrast, when utilizing Multi-GOIRADS, sensitivity > 80% and accuracy above 80% were demonstrated by all three sonographers in classifying omental lesions. Notably, for senior sonographers, the use of Multi-GOIRADS enhanced the PPV to 96.81% and NPV to 72.73% for omental lesions.

### Limitations

The data used in the modeling phase of this study were retrospective, which inherently limits the assessment of omental lesions based solely on static ultrasound images. Variability in the interpretation of lesions among different sonographers may lead to inconsistent descriptions and conclusions. To address these limitations, future studies should incorporate dynamic imaging of omental lesions and provide standardized training for sonographers to enhance their ability to recognize and interpret ultrasound features associated with these lesions. Additionally, the retrospective nature of the data precluded the inclusion of microvascular imaging, resulting in a lack of analysis of microvascular characteristics in our study. Investigating the blood flow characteristics of omental lesions, particularly through microvascular imaging, will be a critical focus of our future research. Furthermore, it is important to acknowledge that the proposed system is relatively complex and requires both high-performance ultrasound equipment and sonographers with sufficient expertise and training. Future efforts should aim to simplify the system, refine the ultrasonic features of omental lesions, and validate its clinical utility through multicenter trials to ensure broader applicability and reliability.

## Conclusion

In this study, a multi-parametric grading system (Multi-GOIRADS) was proposed for risk stratification of malignant omental lesions. The system was based on CEUS parameters, RTE results, and the GOI-RADS. Additionally, the scoring parameters were optimized and adjusted to develop a simplified version, termed sMulti-GOIRADS. Through prospective clinical application, it was found that sMulti-GOIRADS not only reduced the time required for sonographers during diagnosis but also demonstrated excellent diagnostic consistency among physicians with varying levels of expertise. Meanwhile, Multi-GOIRADS exhibited superior diagnostic efficacy in classifying omental lesions. Consequently, Multi-GOIRADS can be utilized in clinical practice for managing ultrasound diagnoses of omental lesions.

## Electronic supplementary material

Below is the link to the electronic supplementary material.

Supplementary Material 1

## Data Availability

No datasets were generated or analysed during the current study.

## Abbreviations

CEUS: Contrast-enhanced ultrasound
RTE: Real-time elastography
OR: Odds ratios
GOI-RADS: Greater Omentum Imaging-Reporting and Data System
CEUS-GOIRADS: Contrast-enhanced ultrasound - Greater Omentum Imaging-Reporting and Data System
RTE-GOIRADS: Real-time elastography - Greater Omentum Imaging-Reporting and Data System
Multi-GOIRADS: Contrast-enhanced ultrasound/ Real-time elastography - Greater Omentum Imaging-Reporting and Data System
ROC: Receiver operating characteristic
ICC: Intraclass Correlation Coefficient
PPV: Positive predictive value
NPV: Negative predictive value
