# Supplementary material for: Innovative optimization of greater omentum imaging report and data system for enhanced risk stratification of omental lesions
Source: Cancer Imaging. 2025 Mar 10;25:28. doi: 10.1186/s40644-025-00848-2 (PMC11892154; doi:10.1186/s40644-025-00848-2)
Supplement: Supplementary file 1 — Supplementary Material 1 [file 40644_2025_848_MOESM1_ESM.docx]

**Innovative Optimization of Greater Omentum Imaging Report and Data System for Enhanced Risk Stratification of Omental Lesions**

Zhiguang Chen ^1^, Liang Sang ^1^, Yuan Cheng ^1^, Xuemei Wang ^1^, Mutian Lv* ^2^, Yanjun Liu* ^1^, ZhiQun Bai* ^1^

1 Department of Ultrasound, The First Hospital of China Medical University, No. 155, Nanjing North Street, Heping District, Shenyang, 110001, Liaoning Province, China.

2 Department of Nuclear Medicine, The First Hospital of China Medical University, No. 155, Nanjing North Street, Heping District, Shenyang, 110001, Liaoning Province, China.

**Materials and methods**

Ultrasound-guided biopsy: Prior to the biopsy procedure, the operator must thoroughly inform the patient of the potential complications and the possibility of negative results. Patients must meet the following criteria: 1) Omental thickness ≥ 15mm; 2) No vital organs located directly behind the omentum or a safe distance (≥ 10mm) separating the omentum from vital organs; 3) Normal coagulation function; 4) Accompanied by a family member; 5) Signed informed consent form; 6) For patients with significant ascites, ascites drainage is necessary before the biopsy.

Supplementary Table 1. General information of 215 patients with peritoneal lesions

|  |  | Number of patients (Num.) | Proportion | Gender | | t/χ^2^ | Age | P |
| --- | --- | --- | --- | --- | --- | --- | --- | --- |
|  |  |  |  | Male | Female |  |  |  |
| Omental malignancy | |  |  | 40 | 126 | 2.18^#^ |  | 0.145^#^ |
| Gynecological disease metastasis | | 94 | 0.5663 |  |  |  | 53.78±15.01 | |
| Gastrointestinal metastasis | | 32 | 0.1928 |  |  |  |  |  |
| Malignant peritoneal mesothelioma | | 12 | 0.0723 |  |  |  |  |  |
| Pseudomyxoma | | 7 | 0.0422 |  |  |  |  |  |
| Metastasis of breast cancer | | 6 | 0.0361 |  |  |  |  |  |
| Lymphoma | | 5 | 0.0301 |  |  |  |  |  |
| Metastasis of pancreas | | 4 | 0.0241 |  |  |  |  |  |
| Metastasis of lung cancer | | 5 | 0.0301 |  |  |  |  |  |
| Intestinal neuroendocrine tumors | | 1 | 0.0060 |  |  |  |  |  |
| Benign lesions of omentum | |  |  | 17 | 32 | **3.167^*^** |  | **0.002^*^** |
|  | Tuberculosis | 38 | 0.7755 |  |  |  | 60.01±11.13 | |
|  | Inflammation | 11 | 0.2245 |  |  |  |  |  |

#Chi square test for gender differences between two groups of patients; * Independent sample t-test to compare age differences between two groups of patients

Supplementary Table 2. General information of 104patients with peritoneal lesions

|  |  | Number of patients (Num.) | Proportion | Gender | | t/χ^2^ | Age | P |
| --- | --- | --- | --- | --- | --- | --- | --- | --- |
|  |  |  |  | Male | Female |  |  |  |
| Omental malignancy | |  |  | 16 | 70 | 1.936^#^ |  | 0.204^#^ |
| Gynecological disease metastasis | | 58 | 67.44 |  |  |  | 53.78±15.01 | |
| Gastrointestinal metastasis | | 12 | 13.95 |  |  |  |  |  |
| Malignant peritoneal mesothelioma | | 5 | 5.81 |  |  |  |  |  |
| Pseudomyxoma | | 4 | 4.65 |  |  |  |  |  |
| Metastasis of breast cancer | | 2 | 2.33 |  |  |  |  |  |
| Lymphoma | | 2 | 2.33 |  |  |  |  |  |
| Metastasis of pancreas | | 1 | 1.16 |  |  |  |  |  |
| Metastasis of lung cancer | | 2 | 2.33 |  |  |  |  |  |
| Benign lesions of omentum | |  |  | 6 | 12 | **2.359^*^** |  | **0.020^*^** |
| Tuberculosis | | 16 | 88.89 |  |  |  | 60.01±11.13 | |
| Inflammation | | 2 | 11.11 |  |  |  |  |  |

#Chi square test for gender differences between two groups of patients; * Independent sample t-test to compare age differences between two groups of patients

Supplementary Table 3. Malignant rate of omental score corresponding to Multi-GOIRADS

| Omental score | 5 | 7 | 8 | 9 | 10 | 11 | 12 | 13 | 14 | 15 | 16 | 17 | 18 | ≥19 |
| --- | --- | --- | --- | --- | --- | --- | --- | --- | --- | --- | --- | --- | --- | --- |
| Benign | 4 | 1 | 0 | 1 | 2 | 1 | 2 | 0 | 2 | 2 | 1 | 1 | 1 | 0 |
| Malignant | 0 | 0 | 1 | 0 | 1 | 0 | 0 | 5 | 3 | 0 | 12 | 6 | 21 | 37 |
| Malignant rate (%) | 0 | 0 | 100 | 0 | 33.33 | 0 | 0 | 100 | 60 | 0 | 92.31 | 85.71 | 95.45 | 100 |

Supplementary Table 4. Diagnostic efficacy of sMulti-GOIRADS

|  |  | Pathological results | | Sen(%) | Spe(%) | Accuracy (%) | PPV(%) | NPV(%) |
| --- | --- | --- | --- | --- | --- | --- | --- | --- |
|  |  | Malignant(86) | Benign(18) |  |  |  |  |  |
| sMulti-GOIRADS | Malignant | 63 | 1 | 73.26 | 94.44 | 76.92 | 98.43 | 42.50 |
|  | Benign | 23 | 17 |  |  |  |  |  |

Supplementary Table 5. Malignant rate of omentum score corresponding to sMulti-GOIRADS

| Omental score | 1 | 2 | 3 | 4 | 5 | 6 | 7 | 8 | 9 | 10 |
| --- | --- | --- | --- | --- | --- | --- | --- | --- | --- | --- |
| Benign | 4 | 1 | 2 | 4 | 2 | 4 | 1 | 0 | 0 | 0 |
| Malignant | 0 | 0 | 2 | 0 | 9 | 12 | 33 | 19 | 8 | 3 |
| Malignant rate (%) | 0 | 0 | 50 | 0 | 81.82 | 75 | 97.06 | 100 | 100 | 100 |


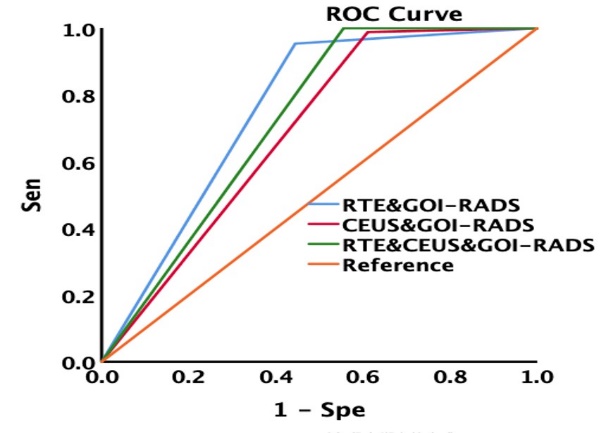


Supplementary Figure 1. Different joint diagnostic strategies of CEUS, RTE, and GOI-RADS. AUC of RTE&GOI-RADS, CEUS&GOI-RADS, and RTE/CEUS&GOI-RADS were 0.755 (95%CI: 0.607, 0.902), 0.689 (95% CI:0.531, 0.847) and 0.722 (95% CI: 0.566,0.878), respectively.


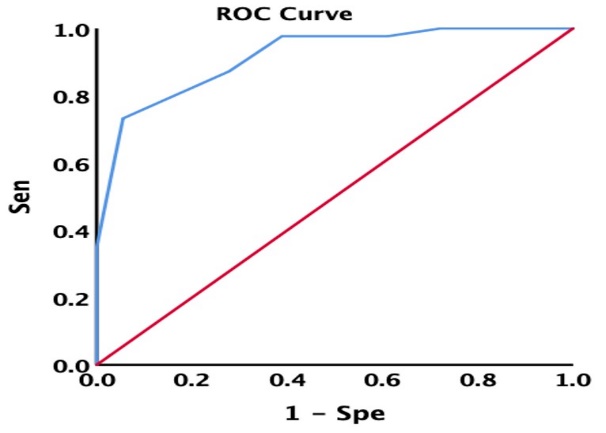


Supplementary Figure 2. ROC of sMulti-GOIRADS.
